# Cytokines and Chemokines in Cancer Cachexia and Its Long-Term Impact on COVID-19

**DOI:** 10.3390/cells11030579

**Published:** 2022-02-08

**Authors:** Santosh Kumar Singh, Rajesh Singh

**Affiliations:** 1 Department of Microbiology, Biochemistry, and Immunology, Morehouse School of Medicine, Atlanta, GA 30310, USA; sksingh@msm.edu; 2 Cancer Health Equity Institute, Morehouse School of Medicine, Atlanta, GA 30310, USA

**Keywords:** cachexia, chemokine, cytokine, SARS-CoV-2

## Abstract

Cancer cachexia remains a serious public health concern worldwide, particularly as cancer rates rise. Treatment is endangered, and survival is reduced, because this illness is commonly misdiagnosed and undertreated. Although weight loss is the most evident sign of cachexia, there are other early metabolic and inflammatory changes that occur before the most obvious symptoms appear. Cachexia-related inflammation is induced by a combination of factors, one of which is the release of inflammation-promoting chemicals by the tumor. Today, more scientists are beginning to believe that the development of SARS-CoV-2 (COVID-19) related cachexia is similar to cancer-related cachexia. It is worth noting that patients infected with COVID-19 have a significant inflammatory response and can develop cachexia. These correlations provide feasible reasons for the variance in the occurrence and severity of cachexia in human malignancies, therefore, specific therapeutic options for these individuals must be addressed based on disease types. In this review, we highlighted the role of key chemokines, cytokines, and clinical management in relation to cancer cachexia and its long-term impact on COVID-19 patients.

## 1. Introduction

Cancer cachexia, a muscle-wasting syndrome, is inextricably linked to the development and progression of tumors such as pancreatic, esophageal, gastric, lung, liver cancers, and others [1]. Cachexia is a multifactorial, pathological disorder that accounts for up to 20% of fatalities in cancer patients. It is characterized by loss of skeletal muscle tissue or sarcopenia, with or without loss of adipose tissue, and frequently accompanied by anorexia, increased metabolism, altered immune function, anemia, and overall decreased quality of life [2]. As a result of the induction of anorexia or reduced food intake, it causes malnutrition, and patients with particular cancers lose the greatest weight. Further, the tumor-host battle for nutrition causes an “accelerated” starving state in the host, resulting in severe metabolic abnormalities and increased energy inefficiency in the host [3,4]. Guidelines for defining the cancer cachexia stages includes unintentional weight loss, then progresses to a more severe, permanent and progressive loss of both muscle and fat, as well as comorbidities such as metabolic and immune system impairments, eventually leading to death, have recently been proposed [2]. It has been demonstrated that non-muscle tissues and organs, as well as tumor tissues, emit soluble substances that act on skeletal muscle to promote wasting [5]. Despite the fact that the hunt for the “cachectic” factor(s) began a long time ago and that considerable scientific and economic resources have been committed to its discovery, however, from the last decades, much progress has been achieved. Proinflammatory and soluble released cytokine mediators within the cancer cell microenvironment contribute to systemic inflammation and act directly on skeletal muscle to cause wasting [5,6]. As a result, the search for mediators and biomarkers has focused on the levels of cachectic substances in plasma [1,7]. 

Cytokines aid in the maintenance of homeostasis. Cytokines can act in three ways: auto-, para-, and endocrine. They control the production of other cytokines and receptors and induce or suppress their production [8]. Multiple cell types rapidly manufacture cytokines in response to varied stimuli, then carried via the systemic circulation [9]. Apart from liver and muscle, it is widely recognized that adipose tissue plays an important role in the pathogenesis of weight loss and metabolic alterations in cancer cachexia. However, the changes in peripheral organs, including adipose tissue, are primarily driven by mechanisms that control the immune response against tumors and the release of specific cytokines, chemokines, and growth factors by immune system cells, allowing them to reach the bloodstream and cause cancer cachexia [10]. Chemokines (which direct cells migration and activation), tumor necrosis factors family (TNF, which regulates inflammatory and immune response), interferons (IF, which regulate antiviral proteins), interleukins (IL, act on various cell types depending on types of IL), transforming growth factors (TGF, which regulate immune cells), growth and colony-stimulating factors (CSF), and other substances are examples of cytokines [8]. Cytokines are not the main cause of disease. On the other hand, cytokines can play a role in the creation of immunopathologic processes and serve as diagnostic markers in specific disorders [9].

Moreover, clinical cytokine uses in treating various conditions has fueled cytokine research. Nonetheless, after the isolation of TNF1, which was then called ‘cachectin,’ the link between immunology and cachexia became a focus of attention [11]. Beutler et al., 1985 discovered that adipocytes exposed to TNF reduce fat uptake by suppressing the production of lipoprotein lipase (LPL) [12]. TNF stimulates adipocyte triglyceride lipase (ATGL)-mediated lipolysis by depleting the ATGL inhibitory protein G0/G1 switch protein 2 in adipocyte cultures [13]. Furthermore, TNF-α, IL-1, IL-6, and IFN-γ impede myoblast development and promote protein loss in myoblast cells via STAT3-mediated NF-kB activation. These findings were confirmed in mouse models, which showed substantial weight loss, adipose tissue depletion, and muscular atrophy in mice transplanted with tumor cells overexpressing TNF-α, IL-1, IL-6, or IFN-γ. Blocking IL-6 or IFN-γ, respectively, alleviated the cachectic phenotype in mice models where cancer cell lines overexpressed it. Notably, in these mice, overexpression of IL-1 and IL-6 lowered overall survival and resulted in more aggressive tumor growth. In a different study, mice treated with pancreatic cancer cells lacking IL-6 expression exhibited less adipose tissue loss and were protected from muscular atrophy [11]. 

The disease, SARS-CoV-2 (COVID-19), which started in Wuhan, China, spread quickly and resulted in a COVID-19 outbreak in just a few months. Previously, an outbreak of severe acute respiratory syndrome (SARS) has been explored; SARS-CoV and SARS-CoV-2 are both positive-strand viruses having the exact origin with 80% genotypic similarity and the same binding affinities for human angiotensin-converting enzyme 2 (ACE2) [14]. The presence of severe forms of SARS-CoV-2 in a subset of individuals demonstrates the immune system’s incapacity to combat the viral infection. SARS-CoV-2 patients have various symptoms and problems, so disease staging was used to identify how far and how severe an illness had progressed. Since, staging systems provide valuable benchmarks for clinical decision-making, inpatient management, prognostication, and treatment selection based on evidence. Like TNM (tumor, lymph nodes, and metastasis) staging in cancer, which has gained global approval for assessing the amount of any given malignancy, COVID-19 were divided into four stages; stage 1: viral entry and replication; stage 2: viral dissemination; stage 3: multisystem inflammation; and stage 4: endothelial damage, thrombosis, and multiorgan dysfunction [15]. COVID-19 stage 4 patients, like cancer patients, have a significant mortality rate. Inflammatory indicators such as C-reactive protein and procalcitonin, and elevated levels of coagulation cascade byproducts, may be useful in forecasting progression to more severe disease. According to Liu et al., convalescent plasma is potentially beneficial against COVID-19 at stages 3 or 4 of the disease; also, found that survival was improved in plasma recipients [16]. In a separate study, increased cytokine levels were associated with COVID-19 disease severity [17]. Those who have high levels of pro-inflammatory cytokines, especially IL-6 and other acute-phase proteins, result in hyper inflammation [18]. 

COVID-19 has been linked to a large rise in proinflammatory cytokines, which has resulted in symptoms similar to those seen in cancer patients with cachexia. A preclinical investigation found that therapies that decrease the host inflammatory response in the early stages of COVID-19 resemble the early phases of cancer-related cachexia can reverse cachexia and increase survival. Anorexia, sudden weight loss, sarcopenia, tiredness, and functional loss, all present in cancer cachexia, have been more common in COVID-19 patients [2,19,20]. Furthermore, cancer patients with COVID-19 may be at an increased risk of sarcopenia and cachexia. 

During hospitalization, fibrinogen and antithrombin levels were significantly low throughout inpatient evaluations, whereas lymphopenia, IL-6, CRP, D-dimer, and ferritin levels were significantly high in non-survivors [18]. Cachexia appears to develop swiftly in those hospitalized for SARS-CoV-2 infection, especially those who have had long stays in the intensive care unit (ICU). According to a recent clinical experiment, NCT04698798, more prolonged ICU stays resulted in considerable skeletal muscle mass and strength losses of 3–4% each day, implying that many mechanisms in SARS-CoV-2 induce cachexia. Moreover, infections with SARS-CoV-2 are more common in cancer patients, who have a higher rate of severe symptoms due to tumor-induced immune suppression [14]. Critical cellular proteins and activities such as intracellular vesicle trafficking, nuclear transport, cytoskeletal integrity, inflammatory signaling, and mitochondrial respiration are severely disrupted by SARS-CoV-2; as a result, contributing to tissue malfunction and increasing local inflammation. According to epidemiological data from the SARS epidemic of 2002 to 2004, myalgias, osteoporosis, muscle weakness, and osteonecrosis were common sequelae in people with moderate and severe forms of the disease [21]. Research has suggested that certain SARS-CoV-2 patients have a significant musculoskeletal impairment, including skeletal muscle, bone, joint and neurological abnormalities [22,23]. The inflammatory response such as C-X-C pattern chemokines, interleukins (IL-1, IL-6), interferons (IFN-γ), and TNF-α cytokines can cause systemic inflammation, affecting practically every organ system, including the musculoskeletal system, in addition to directly infecting cells outside of the respiratory tract [21,24].

The fundamental mechanisms that cause cachexia are unknown, but they appear to be linked, at least in part, to the tumor’s immunological response both locally and systemically [25]. Therefore, a thorough analysis of the gene expression profile of cachexia- inducing factors (CIFs) has the potential to reveal tumor secretome transcriptional patterns, which could assist in explaining the diversity in cachexia prevalence and severity within and across human malignancies [1]. We believe that cytokine levels in the tumor microenvironment and systemic circulation fluctuate significantly amongst tumors and that these changes influence the degree of cancer cachexia observed. This study highlights recent advances in animal models and humans related to using chemokine/or cytokine as a cachexia therapy.

## 2. Local and Systemic Cytokines Associated with Cachexia

Cytokines are the most ubiquitous regulatory mechanism; when released, they control intercellular communication either locally or systematically [9]. Numerous additional cytokines are increased in circulation and local tissue settings in both cancer and infection. The involvement of many of these cytokines in cachexia pathogenesis has not been adequately studied. In the following subsections, we will go through a few cytokines that have been connected to the development of cachexia in cancer and COVID-19 patients.

### 2.1. Chemokines in Cachexia

Chemokines are chemoattractant cytokines that play a role in leukocyte trafficking and determining the metastatic destination of tumor cells. Chemokines bind to their cognate receptors, the majority of which are G-protein coupled receptors and are found in endothelial cells and lymphocytes [26]. Chemokines are classified into two groups depending on their biological activity: those that maintain homeostasis and others that cause inflammation [27]. Recent studies have shown that C-X-C motif chemokine receptor 2 (CXCR2) influence cardiac tissue loss. CXCR2 was shown to be 4.6 times overexpressed in vitro in the early stages of cachexia but only 2.4 times overexpressed in the advanced stages. As a result, CXCR2 expression in myocardial cachexia has positioned the cardiac milieu for substantial changes [28]. 

In a separate study of colorectal cancer (CRC), Scheede-Bergdahl et al. compared serum concentrations of four pro-inflammatory factors (IL-6, IL-1, Chemokine (C-X-C Motif) ligand 8(CXCL8)/IL-8, and TNF-α) in advanced-stage cancer patients. They discovered that IL-1 levels linked more strongly with clinical features than IL-6 levels [29]. Studies evaluating tumor-associated macrophages (TAMs), the most abundantly represented inflammatory cells in the CRC, were the subject of another survey [30]. TAMs play a dual role in the progression and development of CRC. Various phenotypic proportions of M1/M2 TAMs have been described in CRC, with varying degrees of association with CRC patient survival. According to Ong et al., TAMs were pro-inflammatory and decreased tumor cell growth and release cytokines (e.g., IL-6 and IFN-γ) and chemokines (e.g., CXCL8/IL-8 and CCL2) that attract T-cells and boost type-1 Tcell response. As a result, TAMs induced tumor-suppressive effects via T cells [31]. In conclusion, the M1/M2 macrophage ratio in TME and the immune response mediators released by these cells play a role in CRC-associated cachexia (mainly IL-1 and IL-6). In addition, some researchers believe that a decrease in the number of neutrophils, macrophages, and mesenchymal progenitors near the injury site causes worse muscle regeneration in a mouse model of cachexia, which could be driven in part by a decrease in chemokine (CCL2-CCL5) production [32]. 

Furthermore, individuals with pancreatic cancer who had their tumor resected had low specified pro-inflammatory cytokines (IL-6, IL-1, IFN-γ, and TNF-α), which were not linked to cancer cachexia. Only the monocyte chemoattractant protein 1 (MCP-1) was found to be higher in treatment-naive cachectic patients than in those who did not have the condition. As a result, it was recommended as a potential cachexia biomarker in humans and animals to diagnose early cachectic states and distinguish cancer-induced cachexia [33]. 

By studying the expression profiles of secreted genes in diverse human malignancies, Freire et al. revealed pathways and mediators with a potential role in cachexia inside the tumor microenvironment [1]. They discovered that cytokine- and chemokine-related pathways were overrepresented in tumor types linked to the condition. Cachexia prevalence and weight loss were linked to the number of elevated genes. CXCL8 expression was high in six tumor types with a high frequency of cachexia (STAD, ESCA, COAD, PAAD, READ, and HNSC) [1]. CXCL8 has also been connected to weight loss in pancreatic cancer, stomach cancer, and colorectal cancer patients [34,35,36]. Pancreatic cancer patients lose an average of 13.7 kg, associated with the overexpression of 14 cachexia-inducing factor genes (CCL2, CXCL8, IL-1B, IL-6, IL-15, TNFSF10, TNFSF11, CSF1, CSF2, HGF, LIF, TGFA, PDGFB, and FGF2). However, when compared to normal tissues, prostate cancer (PCa) patients lose roughly 1.7 kg of weight and do not upregulate cachexia-inducing factor genes. This demonstrates the discrepancy in average weight loss and the occurrence of cachexia among cancer patients [1]. Overall, these investigations revealed the expression of cancer-related tumor cachectic factors genes, which may explain why some cancer types are more prone to cachexia. 

Numerous studies have demonstrated the significance of adipose tissue in the onset and maintenance of systemic inflammation [37,38]. Adipose tissue releases a number of bioactive chemicals, including IL-1, IL-6, TNF-α, and CCL2, which can reach the bloodstream and enhance crosstalk with other tissues [39]. Increased CCL4 gene expression was detected in obese mice, along with an increase in infiltrating leukocytes. Furthermore, cachectic patients had higher TNF-α, CCL2, and CCL3 protein expression than weight-stabilized cancer patients. Even CCL3 protein levels in the patients’ tumors have a positive association with the expression of pro-inflammatory IL-1 protein. Besides, a positive association between CCL4 and TNF-α was discovered in the subcutaneous adipose tissue [40]. These findings support the idea of a complex and dynamic interaction between the tumor and peripheral tissues, with infiltrating immune cells playing a key role. 

Cachexia, a wasting syndrome associated with a caloric deficit, is probable in cancer patients. Relative hypo ketonemia occurs when mice are starved of calories, causing glucocorticoid levels to rise. As a result, this hormonal stress response suppresses multiple intratumoral immune pathways [41]. Flint et al. investigated the expression of a panel of eight genes involved in CXCR3-dependent chemotaxis, T cell presence, and T cell effector activities. All of these immunological events were reduced in cachectic mouse tumors. Furthermore, the expression of this immune gene panel was reduced in food-restricted pre-cachectic C26-bearing animals in a similar way to cachectic C26-bearing mice. Also, the decreased expression of CXCL9, CXCL10, and CXCL11 shows that the intratumoral myelomonocytic cell may be the most significant cellular target of increased glucocorticoids. In these cells, deletion of the glucocorticoid receptor NR3C1 abolished the glucocorticoid-dependent inhibition of CXCL10 and other chemotactic proteins [41,42]. Therefore, we hope that a greater understanding of the role of chemokines, cytokines, immunotherapy, and metabolic dysregulation during cachexia will facilitate the development of new cancer therapies and biomarkers.

### 2.2. Cytokines in Cachexia

Cytokines are small proteins released by cells that regulate cell contact and communication in unique ways. Cytokines can be pro-inflammatory or anti-inflammatory, and they can affect the cells that release them and nearby cells and, in certain situations, distant cells. They are frequently produced in a cascade, with one cytokine stimulating the production of additional cytokines by its target cells [8]. Cytokines can work together or against each other. The long-term production of cytokines is increasingly recognized as a major contributor to diseases such as cancer, chronic infections, and cachexia [43]. 

According to a review, cachexia is a distressing and complex condition that affects 50–80% of cancer patients, depending on the tumor type. The occurrence of cachexia in cancer patients is influenced by the patient’s response to tumor progression, which includes the activation of the inflammatory response and energy inefficiency involving the mitochondria. Inflammation is a common occurrence in cancer patients, caused by the release of cytokines, chemokines, and other inflammatory mediators by tumor cells and activated immune cells [44]. Additionally, previous research has found that when the gut barrier is disrupted, the formation of lipopolysaccharide (LPS) and other bacterial toxins increases immune cell cytokine synthesis and release [45]. Furthermore, myostatin [46], a protein linked to skeletal muscle loss that works through activin receptor type IIB (ACTRIIB)-mediated signaling, and glucocorticoids [47] have recently been discovered to be a determinant of inflammation-driven atrophy, suggesting that they may play a role in cancer cachexia etiology.

In addition to inflammatory mediators linked to muscle atrophy, cytokines stimulate transcription factor activation, contributing to cancer’s metabolic abnormalities [44]. Inflammatory mediators such as IL-1, IL-6, TNF-α, and IFN-γ are generated in cancer cachexia, resulting in increased energy expenditure, decreased appetite, and muscular atrophy [48]. These substances provide leptin-like signals to the hypothalamus in the brain [49], increasing corticotropin-releasing hormone expression while reducing ghrelin [50], resulting in appetite control. Knowing that a move forward on the defined path of cachexia, a weight loss of more than 5% in the previous 6 months, or a weight loss of more than 2% in patients with a BMI of less than 20 Kg/m^2^, Jo et al. investigated the predictive value of cachexia in advanced NSCLC patients (133 patients) treated with PD-L1 inhibitors (pembrolizumab); they looked at the levels of pro-inflammatory cytokines and appetite-related hormones in the blood serum, which are linked to the pathogenesis of cancer cachexia. In the end, inflammatory mediators and leptin levels were all found to be substantially linked with cachexia [48]. 

Further, a separate investigation identified the Hypoxia-inducible factor (HIF) as the primary event driving metabolic alterations in cancer cachexia syndrome. HIF regulates macrophage metabolism, causing them to become M1-polarized and produce proinflammatory cytokines that keep the inflammatory state going for a long time, shared in cancer cachexia patients. In conclusion, the presence of persistent inflammation mediators in the tumor microenvironment transforms the first encounter between cancer cells and immune cells into a systemic disease characterized by specific symptoms connected to tissue damage [10,51]. 

In cancer patients, cachexia syndrome is linked to a greater mortality rate. According to recent data, it has a significant negative influence on patients’ quality of life [52]. This study covers important pro-and anti-inflammatory cytokines and putative underlying mechanisms and their relevance to cancer cachexia.

#### 2.2.1. Interleukins

A study demonstrated that cytokine mediators and growth factors (IL-1, IL-4, IL-6, TNF-α, IFN-γ, TGF-β) regulate the control of the central nervous system in cancer patients, despite the continual loss of skeletal muscle induced by a number of processes [53,54]. IL-1 has been studied as a regulator of local and systemic inflammation in innate and adaptive immunity and a therapeutic target for cancer cachexia, resulting in an antibody (Bermekimab) formation. Binding to the IL-R1 receptor causes the overexpression of a number of other inflammatory cytokines, including IL-6 and TNF-α. It has been proven in animal and human models to promote carcinogenesis by activating nuclear factor B (NFB) regulatory genes, enhancing cytokine and chemokine release [55].

Additionally, IL-6 is the primary regulator of the acute-phase response in cachectic individuals. Growing evidence suggests that IL-6 plays a role in cancer metastasis. In tumor cell lines, overexpression of IL-6 increased myeloid cell recruitment, angiogenesis, and metastasis [56]. Proteolysis, mediated mostly by IL-6, results in the release of amino acids during inflammation. This impact is likely an attempt to reactivate mTOR and effectively sustain increased energy demand through -oxidation. Importantly, IL-6 is a facilitator of cancer anorexia and the primary inducer of cancer anemia, and both states inhibit the mTOR axis, resulting in a vicious loop that accelerates muscle wasting [10].

Chronic IL-6 exposure causes wasting in skeletal muscle via inducing proteasome and autophagy protein breakdown pathways. Furthermore, IL-6 is indirectly linked to the activation of AMPK and NF-kB, and TLR4. IL-6 can impair cachectic patient recovery by targeting skeletal muscle, liver, gut, and adipose tissue. Blocking IL-6 and related signals have been shown to slow the progression of cachexia in several animal cancer models [57]. In the cachectic Apc^Min^/+ mice, inhibiting IL-6 signaling by systemic injection of IL-6R Ab decreases body weight and muscle loss without restoring MPS; mTOR signaling was also downregulated in mice who consumed glucose and exercised [57,58,59]. In addition, IL-6 functions through activating leukocytes, specifically by preparing macrophages to adopt an anti-inflammatory phenotype. Muscle atrophy, cancer, and the development of liver acute phase proteins have all been linked to IL-6 [60]. Counteracting anorexia and anemia and limiting the action of inflammatory cytokines, particularly IL-6, should be part of any strategy to correct this illness [10]. 

#### 2.2.2. Tumor Necrosis Factors

Tumor necrosis factor (TNF-α) is involved in all critical processes of cancer cachexia, including adipose tissue loss, skeletal muscle loss, changes in glucose, protein, and systemic inflammation [61]. This cytokine, TNF-α, was first blamed for these changes in cachexia. However, it has become obvious from many investigations that TNF’s activity can only be understood in the presence of other cytokines, some of which have activities that are at least as important as TNF-α in causing cachexia [43]. When nude mice were implanted with Chinese Hamster Ovary cells (CHO) transfected with the human TNF gene, they developed anorexia, progressive wasting, and mortality [62]. TNF-α, found in the tumor-bearing rats, raises corticotrophin-releasing hormone (CRH) and lowers food consumption [63]. It was observed that long-term therapy with recombinant TNF-α causes protein redistribution and a significant decrease in muscle protein content [64]. Furthermore, transgenic mice lacking the TNF-α-receptor protein type I (TNFR1) were transplanted with Lewis lung carcinoma; they showed less muscle wasting than wild-type mice, despite the fact that both had similar TNF levels in their serum [65]. In addition, TNF-α plays an important role in altering carbohydrate and lipid metabolism and insulin resistance, which has been identified as essential contributors in cancer cachexia metabolism [66,67]. According to Borst et al., TNF-α overexpression leads to the etiology of insulin resistance. Insulin resistance is a hallmark of the metabolic syndrome associated with obesity, TNF-α, IL-6, adiponectin, resistin, and free fatty acids, all of which are contained in visceral fat, may have a role in the development of insulin resistance [67].

Changes in lipid catabolism, presumably, lipogenesis, cause severe lipid loss in cancer cachexia. Increased plasma levels of fatty acids, TNF-α, IL-1, IL-6, glycerol, and lipid-mobilizing factors (zinc-2 glycoprotein-1) support the idea that increased triacylglycerol (TG) degradation may contribute decisively to cachexia. And disrupting fat catabolism could prevent cancer-associated cachexia from starting or progressing [68]. TNF is a protein that plays an important role in inflammation and disease. TNF receptor 1 (TNF-R1)- induced NF-kB interaction dysregulation causes cell proliferation and chronic inflammatory disease [69]. Mondrinos et al. investigated the spatiotemporal distribution of tumor-derived TNF using a 3D microphysiological model of lung cancer cachexia. Accordingly, TNF diffusive transport from the spheroid chambers gradually increased TNF levels in the muscle compartment. The muscle tissues in the device had dramatically increased nuclear translocation of p65/nuclear factor B (NF-kB), which was in line with the computational predictions and represented the proinflammatory character of the muscle injury in the model [70]. Therefore, TNF neutralization could reduce inflammation and inflammatory- related diseases.

#### 2.2.3. Transforming Growth Factor

Many disorders, including cancer, disrupt the transforming growth factor (TGF) signaling pathway. This pathway possesses tumor-suppressor activities in healthy cells and early-stage tumor malignancy; however, it promotes carcinogenesis, drug resistance, and metastasis in the delayed stage [71]. TGF-β is an appealing target for cancer therapy because of its critical involvement in tumor growth. A study found that tumor remodeling causes an imbalanced inflammatory cytokine profile, extracellular matrix (ECM), and revascularization, most likely due to TGF-β [72]. The researchers were able to show in mice model that cancers metastasis to bone cause osteolysis release of TGF-β from the extracellular matrix of the bone into the general circulation. Also, the circulating amounts of TGF-β activate a SMAD3 signaling pathway in both proximal and distal skeletal muscles [73]. TGF-β signaling through SMAD3 transcriptionally promotes the NADPH oxidase 4 (Nox4) gene, which oxidizes various proteins, including ryanodine receptor5 (RyR). As a result, tumors that migrate to bone and release TGF-β result in poorer muscular force production regardless of whether muscle mass and body weight are reduced [74]. Also recognized that the activated conventional SMAD2/3 pathway promotes muscle fibrosis and ubiquitin ligase-mediated proteolysis while inhibiting Akt/mTOR-mediated tissue growth [75].

According to recent investigations, protein breakdown has been linked to muscle depletion in cachexia. Various sophisticated signaling pathways such as ubiquitin-proteasome and autophagy processes are required to activate the muscle protein breakdown machinery in response to cancer. Autophagy has recently been demonstrated to have a role in the pathophysiology of muscle wasting [76]. Liu et al. discovered that p38 MAPK promotes autophagy activation in cachectic mice [77]. Further, TGF-β has been demonstrated to activate the p38β MAPK pathway [78]. The key function of p38β MAPK activates C/EBPβ, which is essential for cancer-related muscle atrophy [79]. p38β MAPK plays a regulatory function in many signaling stages that are rate-limiting for muscle catabolism [77]. This research also shows that p38β MAPK-mediated phosphorylation of Ser-12 on p300 is essential and sufficient for cancer-induced muscle atrophy. In tumor-bearing mice, systemic injection of an FDA-approved kinase inhibitor, nilotinib, at a modest dose, decreased p300 activation 20-fold more potently than SB202190. This p38/MAPK inhibitor is reduced muscle wasting and extends longevity. Therefore, it was proposed as a viable treatment for cancer cachexia [78].

### 2.3. SARS-CoV-2 (COVID-19) Induced Cytokine Storm in Muscle Wasting and Their Clinical Management

Systemic inflammation, hypoxemia, muscle fiber loss, metabolic changes, malnutrition, and exercise intolerance are common in patients with severe COVID-19 that contribute to a significant portion of weakness and weariness in patients [80]. Researchers had to go profoundly into the molecular and cellular basis of SARS-CoV-2-induced immune responses to discover novel biomarkers, predictive tools, and a new therapeutic option. Once the virus has entered the body, it begins multiplication, and a chain of events occurs that causes epithelial and endothelial cell death and vascular leakage [81]. As a result of this event, it stimulates to release of various pro-inflammatory cytokines and chemokines like C-reactive protein, IL family (IL-6, IL-10), ferritin, TNF-α, fibroblast growth factor, NF-kB, interferons (IFN)-induced protein 10 (IP-10), and others that are responsible for SARS-CoV-2’s aggressive inflammation [82,83]. In fact, research has found that patients with COVID-19 have weight loss and cachexia, which are associated with elevated inflammatory markers (CRP), reduced renal function, and a longer duration of this disease. Further, muscle atrophy is also caused by IL-6 driven stimulation of muscular STAT3/NF-kB signaling and proteolysis mediated by the ubiquitin-proteasome system in skeletal muscle. In a preclinical ovarian cancer model examining the role of IL-6 in cancer-related cachexia, they discovered severe weight loss and progressive muscle wasting due to altered metabolism and elevated levels of IL-6, p-STAT3, and reduced p-Akt levels [84]. Also, prolonged exposure to high IL-6 in patients with severe COVID-19 may cause serious muscle wasting, which worsens organ failure, including respiratory failure, as reported in other severe viral infectious diseases [18,85].

According to Meyer et al., computed tomography (CT) can be utilized as a biomarker to define low skeletal muscle mass (LSMM) and visceral fat areas in COVID-19 patients. The meta-analysis discovered that 33.6% of patients were with LSMM, extended bed rest, and systemic inflammation was at risk of SMM [86]. Additionally, aged patients with primary sarcopenia are more prone to related muscular wasting than those who do not [87]. Furthermore, visceral fat is considered harmful in a number of disorders because it produces pro-inflammatory cytokines that are transported directly into the bloodstream, triggering cytokine storms. The ACE2, which is overexpressed in several tissues, including the visceral fat, is exploited by the SARS-CoV-2 virus as a gateway into the body, supporting the link between the severity of COVID-19 infection and fat distribution; therefore, a high visceral fat level is considered as a prognostic factor in COVID patients [86,88]. 

Skeletal muscle produces a variety of soluble anti-inflammatory and immunoprotected substances known as myokines, which may alleviate the disease’s aggravated inflammation. Therefore, it could be used in COVID-19 clinical outcomes [89]. Gil et al. assessed 196 hospitalized patients for vastus lateralis cross-sectional area using ultrasonography and concluded that low muscle mass contributes to higher skeleton muscle loss among COVID-19 patients due to increased demands of tissues such as liver and immune cells [90]. Those patients with weak muscle strength are more vulnerable to stress factors, which could be the case of COVID-19 [91,92]. Other studies have found that with COVID-19 treatment, 43% of study participants lost ≥5% of their body weight, and 25% of participants lost ≥10% of their body weight. A strong link was seen between impaired eating function and the switch to parenteral feeding in subjects who lost 10% of their body weight. COVID-19 infection also placed the subjects of this trial at risk of malnutrition [93].

As previously reported in the literature, increased circulating levels of pro-inflammatory cytokines and chemokines (CXCL10 and CCL2) are associated with pulmonary inflammation and extensive lung involvement in SARS patients, like middle east respiratory syndrome (MERS-CoV) infection [94]. Similarly, patients infected with COVID-19 have increased levels of these factors strongly suggested that T helper-1 (Th1) cell activity had been activated, so-called “cytokine storm”. Furthermore, large numbers of pro-inflammatory cytokines (IFN-γ, IL-1, IL-6, IL-12, IL-18, IL-33, TNF-α, TGF-β) and chemokines (CXCL10, CXCL8, CXCL9, CCL2, CCL3, CCL5) are released by immune effector cells in this setting, which sustains the abnormal systemic inflammatory response [95]. All these factors impair the bone and joint tissue physiology in those patients [96]. Also, those COVID-19 patients in hospitalization had higher CCL2, CXCL10, and TNF-α than those who had a non-hospitalized and less severe condition.

**Figure 1:**
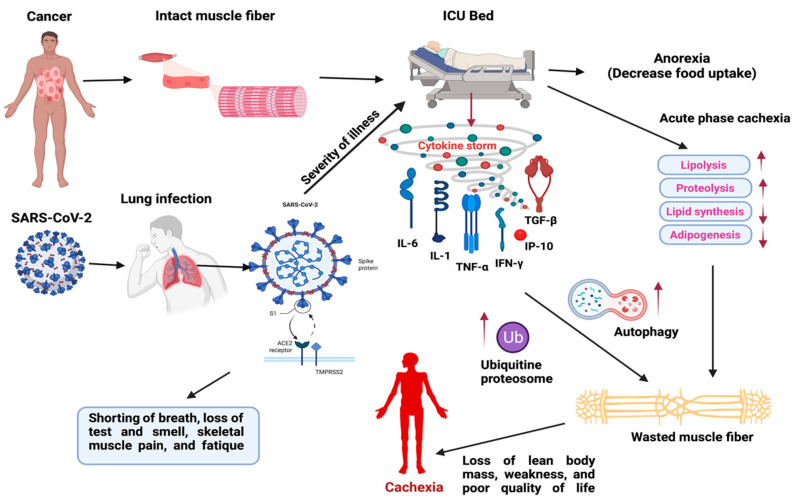
Representative model displays the effect of cytokine storm in cancer or COVID-19-related cachexia. Physical inactivity increases systemic inflammation in hospitalized cancer or COVID-19 patients. Increased levels of these cytokines (IL-1, IL-6, TNF-α, IFN-γ, IP-10, TGF-β) impair hypothalamic appetite control and cause anorexia. At the same time, the ubiquitin-proteasome or autophagy triggered by pro-inflammatory mediators or proteins exacerbates muscle degeneration. Furthermore, during the acute phase of cachexia, adipose tissue is lost either by increased lipolysis or decreased lipid synthesis or adipogenesis. As a result, patients with infrequent skeletal muscle contractions feel tiredness, weakness, and a lower quality of life. (Created with BioRender.com).

Cachexia can be caused by various conditions and illnesses that lead to a decline in nutritional status. Despite the fact that SARS-CoV-2 is contagious in the general population, most hospitalized patients are older or have chronic underlying illnesses. Furthermore, it causes gastrointestinal symptoms, which further jeopardizes older people’s nutritional status, and those patients are at major risk of malnutrition [82,97]. Some COVID-19 patients have shown considerable musculoskeletal damage to a preliminary study. Although long-term follow-up studies have not yet been completed in terms of cachexia in those COVID patients who had prolonged hospitalization, however, changes in cytokine expression are found critical for COVID-19. CXCL10, IL-17, and TNF-α, among the cytokines increased by COVID-19, have established roles in osteoblast proliferation and osteoclastogenesis, resulting in a reduced bone mineral density. Similarly, IL-1b, IL-17, and TN-Fα worsen degenerative tendon disorders, whilst IL-1b, IL-6, and TNF-α have been linked to osteoarthritis development [21]. In addition, a chemokine, CXCL10, is found relevant in the prediction of worse outcomes due to infection [98], since its expression pattern in COVID-19 patients differs from that seen in individuals with other types of viral infections [83]. Therefore, an optimal characterization and a deep understanding of cytokines are required to determine the effect of SARS-CoV-2 infection on muscle wasting. The effect of cytokine/ chemokine storm in cancer or COVID-19-related cachexia is shown in Figure 1.

Today, more scientists are beginning to believe that the development of COVID-19 related cachexia is similar to cancer-related cachexia. Nevertheless, there are no standards for preventing or treating the acute phase of SARS-CoV-2 cachectic patients. Nutritional advice, laboratory testing such as albumin levels, appetite stimulants such as megestrol acetate and glucocorticoids, or a combination of pharmaceutical therapies aimed at underlying pathophysiology may be appropriate in some cases [99,100]. During viral infections, low levels of micronutrients such as vitamins A, D, E, B6, B12, zinc, and selenium have been linked to poor clinical outcomes that should be evaluated during the SARS-CoV-2 therapy [101,102,103]. Natural substances with anti-inflammatory and anti-tumorigenic properties, in addition to micronutrients, have also been discovered to play a significant impact in cancer or COVID-19 -related cachexia [104]. 

Furthermore, in a clinical trial (NCT04698798), Frank et al. studied the effects of SARS-CoV-2 infection on muscle wasting. Considering cachexia in SARS-CoV-2 patients, by adhering to the angiotensin ACE-2, the virus can first interact with muscle cells. According to in vitro studies, the virus can cause myofibrillar fragmentation into individual sarcomeres and loss of cardiomyocyte nuclear DNA. They discovered similar observations during autopsies. At the molecular level, however, the effects of SARS-CoV-2 infection on skeletal muscle cells are unknown. Moreover, patients infected with the SARS-CoV-2 virus have been found to have higher levels of C-reactive protein and other inflammatory cytokines. However, up to 19.3% of patients present with myalgia and elevated levels of creatine kinases (>200 U/L), suggesting skeletal muscle injury [105]. These are not the only ones; other factors, including anorexia, bed rest, immobilization, and mechanical ventilation, all impact skeletal muscle mass in ICU patients. Therefore, a better knowledge of the effects of SARS-CoV-2 infection on acute skeletal muscle wasting is required in patients admitted to the ICU. 

Concerning cachexia and muscle loss, Anker et al. reported the data on 589 patients in ICU or intermediate care after infection with COVID-19 [20]. Those patients displayed significant weight loss after infection, also found to be correlated with raised C-reactive protein level, prolonged stay, and impaired renal function. A clinical trial (NCT 0435059333 phase 3) of SGLT2 inhibitors (dapagliflozin) started during the hospitalization of a COVID patient, in addition, this virus has the potential for causing fibrosis in several parts of the body, including the lungs, and heart, which can lead to chronic wasting [20]. 

## 3. Clinical Management of Cancer Cachexia

Clinical research led to the licensing of several pro-inflammatory cytokines, which have anti-tumor effects in treating numerous cancers, despite their limited efficacy in animal models. Antibodies that suppress immunological checkpoints and chimeric antigen receptor T cells have recently been added to clinical practice. A surge in clinical trials examining the safety and efficacy of cytokine-based medications, both as single agents and in conjunction with other immunomodulatory drugs, has resulted from a growing interest in the anti-tumor capabilities of cytokines [106]. 

Two pro-inflammatory cytokines, IL-2 and IFN-γ, showed some therapeutic benefit and were approved by the Food and Drug Administration (FDA) to treat a variety of cancers, including non-Hodgkin lymphoma, hairy cell leukemia, renal cell carcinoma, metastatic melanoma, and Kaposi’s sarcoma [106]. However, low response rates and severe toxicity were the significant pitfalls with these high-dose treatments; therefore, these cytokines have been consigned to the sidelines in favor of targeted therapy and immune checkpoint inhibitors in clinical practice [107,108]. Although these cytokines are not drugs to treat cachexia, they can provide a unique tool to formulate various survival responses for cachectic patients in clinical trials. Just a few medicines have shown promise in more significant phase III trials. The most notable clinical effects have been demonstrated by combining thalidomide, a glutamic acid derivative with immunomodulatory and anti-inflammatory activities, and a natural human IgG1k antibody (MABp1) against IL-1α [109].

Cachexia and tiredness are being treated with ALD518, a humanized monoclonal antibody that binds with high affinity to IL-6 [57]. Indeed, in phase II randomized trial, ALD518 treatment was well tolerated with fewer side effects and reduced lean body mass loss, fatigue, and anemia in advanced cancer (NSCLC) [110]. Furthermore, weight loss has long been recognized as a marker of poor survival in advanced gastric cancer. Only a few research, have looked into the weight loss that occurs during chemotherapy. Since patient survival is closely associated with lean body mass loss, ideal chemotherapeutic drugs or anti-cytokine therapy should have no remaining lesion in cancer patients. In this context, Lu et al. studied the effective concentrations of macrophage inhibitory cytokine-1 (MIC-1), which identified as a possible etiological component in anorexia and weight loss in patients with advanced gastric cancer during treatment [111]. Moreover, tumor cells or immunological cells release chemical compounds that act directly on muscle, producing atrophy and/or anorexia. MIC-1/GDF15, a TGF-superfamily cytokine generated in large amounts by cancer cells, appears to be a crucial contributor. Patients with anorexia/cachexia syndromes with elevated cytokine levels are a target for MIC-1/GDF15 therapy, and such drugs are in preclinical development. On the other hand, the long-term implications of MIC-1/GDF15 could be beneficial in treating abnormal obesity and its consequences [112]. 

The fundamental stage in cachexia is most likely the systemic inflammatory response, followed by increased production of pro-inflammatory cytokines like TNF-α. Several functional single-nucleotide polymorphisms (SNPs) within the TNF-α gene were previously discovered and reported as cancer-related genetic alterations in a case-control research NCT04131478. TNF-α is primarily considered to enhance acute immunological responses in cancer immunotherapy [113]. TNF-α has been proven in animal models to have a negative impact on immunotherapies that target the PD-1 pathway [114]. The suggested mechanism underlying anti-PD-1 antibody therapy-induced TNF-α mediated the secondary checkpoint component TIM-3 in CD8+ T cells. However, in a phase I clinical trial (NCT03293784), the safety of a combined drug of nivolumab, ipilimumab, and a TNF-α blocking antibody (infliximab or certolizumab) is being assessed. 

One of the clinical trials investigating a TNF inhibitor (Etanercept, a recombinant fusion protein of TNF-α type II receptor) to treat cancer anorexia/weight loss syndrome in patients with solid tumor malignancies did not improve the outcomes of weight, appetite, or survival. The fact that (1) numerous additional cytokines function in concert to mediate this illness, and (2) a significantly higher level of TNF-α inhibition is required to achieve therapeutic efficacy could be one explanation for the trial’s unfavorable results. Even though the present study had a small sample size, it shows that additional research into this syndrome with this drug at this level is unlikely to provide beneficial results [115]. Similarly, a placebo-controlled study was conducted to investigate the therapeutic benefit of infliximab given with gemcitabine in patients with advanced-stage (II-IV) pancreatic cancer. Compared to placebo, the data demonstrate no significant differences in safety or efficacy [116]. According to new research, an antibody against fibroblast growth factor-inducible 14 (Fn14), a receptor for the TWEAK cytokine, increased lifespan in C26 tumor-bearing mice by preventing tumor-induced weight loss, which could be a promising treatment option for cachectic patients [117].

A large, randomized phase 3 trial displayed that a combination of common anti-cachectic medicines is more effective than taking just one alone. Three hundred thirty-two patients with advanced cancer and cachexia were studied by Mantovani et al., who were randomly assigned to one of the five treatments: (1) megestrol acetate (MA) or medroxyprogesterone, (2) eicosapentaenoic acid, (3) L-carnitine. (4) thalidomide, or (5) a combination of the first four medicines. The findings demonstrated statistical differences between the treatment arms. A post hoc analysis revealed that arm 5 outperformed the others for all primary and secondary outcomes, including physical activity, Glasgow Prognostic Score, and IL-6 [118]. Since then, other studies have found that combination therapies are more beneficial than monotherapy. Medications or treatments that are now being investigated in animal models and phase I and phase II clinical research, such as IL-6, ghrelin, or steroid androgen-receptor (AR) modulators, could be promptly translated into phase III clinical trials [118].

In a randomized, phase 2 trial (NCT01505530), Golan et al. evaluated LY2495655 (anti-myostatin antibody) plus standard-of-care chemotherapy in stage II-IV pancreatic cancer patients utilizing cachexia status as a stratification variable in 125 individuals [119]. This combination was failed to yield further therapeutic benefits in a Phase 2 trial of patients with advanced pancreatic cancer (the primary endpoint was overall survival). In a separate systematic analysis of clinical trials, researchers looked at the effectiveness of pharmacological therapies for cachexia in cancer patients. The impact of pharmacological therapy on changes in weight or lean body mass in cancer patients was the primary outcome. Anamorelin (50 or 100 mg/day for 12 weeks) and Enobosarm (1 and 3 mg/day) showed good benefits in cancer patients, with considerable weight loss relative to baseline [120]. As a result, combining medicines with anti-cytokines may provide optimal benefit to target numerous mechanisms underlying cachexia. Because of limited improvement in cancer cachexia prognosis, recent clinical trials combine the inhibitor with other drugs to improve patient outcomes, including therapeutic effectiveness and fewer side effects. Some of the ongoing or completed clinical trials overview on cancer, or COVID-19 related-cachexia are shown in Table 1.

## 4. Conclusions

Cachexia is a multimodal clinically relevant symptom in cancer characterized by skeletal muscle or adipose tissue loss, loss of appetite, impaired anticancer treatment tolerance, and poor quality of life. Patients with cachexia reported higher rates of anorexia, inflammation, tiredness, and worse overall survival than patients without cachexia. While many molecular events in the cytokines or chemokines pathway play roles in tumor initiation and progression, invasion, migration, and metastasis, the steps involved in cachexia are not well understood. It is essential to investigate these underlying complex molecular events. Despite advancements in detection and treatment, effective pharmacological therapies to treat cancer-cachexia are lacking. As a result, a breakthrough in cytokine research will help researchers better grasp the factors that drive the start and progression of this multisystem disease. Recent research, including experiments on anti-cytokine drugs targeting one or more molecules (e.g., IL-1, IL-6, TNF-α, TGF-β, and others), has been studied in phase I and II clinical trials for the treatment of cachexia [109,119,120]. In addition, COVID-19 patients have shown considerable musculoskeletal damage in the study, possibly due to various cytokines’ involvement and prolonged hospitalization. Because of the complicated mechanisms underlying cachexia, a multidisciplinary treatment plan incorporating pharmacological drugs, anti-cytokine/ chemokine therapy, and a combination of other factors such as movement practices along with nourishment is required. Such activities can improve treatment adherence, prognosis, and overall survival in patients with cancer or COVID-19 -induced cachexia.

## Figures and Tables

**Table 1:** Clinical trials overview on cancer or COVID-19-related cachexia.

Patients	Intervention	Status	Identifier Numbers	Initiation Date	Outcomes
Cachectic or non- cahectic Egyptian patients with lung, pancreas, or colon cancer	Pharmacogenetic testing for TNF-α	Unknown	NCT04131478	October 2019	TNF-α -1031T/C and 308 G/A as a cachexia risk factor and SOCS1, TAB2, and FOXP3 biochemical markers
Pancreatic cancer	Xilonix (IL-α antagonist) + Onivyde and 5-FU Drug	I	NCT03207724	July 2017	Increased LBM, WS, QOL
Non-Small Cell Lung Cancer	Anamorelin HCl	III	NCT01395914	July 2011	Improved BW and symptom burden
Cachexia and anorexia in patients with advanced cancer	Etanercept (TNF-α inhibitor)	III	NCT00046904	January 2003	No inhibition of cancer anorexia/weight loss syndrome [115]
Pancreatic cancer	Infliximab (TNF-α inhibitor) + Gemcitabine	II	NCT00060502	May 2003	No difference in LBM
Lung or Pancrease	BYM338 (mAb against activin receptor type2B)	II	NCT01433263	August 2011	Increased thigh muscle volume
Non-small cell lung cancer	GTx-024 (Enobosarm)	III	NCT01355484	May 2011	Improvement in LBM
Non-small cell lung cancer	ALD518 (anti-I-L6 mAb)	II	NCT00866970	March 2009	Raised Hb, improved cachexia [121]
Pancreatic cancer	LY2495655 (anti-myostatin- mAb)	II	NCT01505530	February 2012	PFS, OS [119]
Colorectal cancer	MABp1 (IL-1α inhibitor)	III	NCT02138422	May 2014	Increased LBM, less pain, fatigue, or anorexia [122]
Advanced cancer	OHR/AVR118 (immunomodulator that targets TNF-α and IL-6)	II	NCT01206335	September 2010	Improved WG, decreased fatigue, improved appetite, and strength
Advanced cancer	Cannabis capsules	I	NCT02359123	February 2015	Decreased TNF-α level, stable weight, the weight increase of ≥10%, improved appetite, reduced pain and fatigue
Advanced tumors	Thalidomide (anti-TNF-α)	II	NCT00027638	January 2003	Attenuates loss of weight and LBM [123]
Skeletal muscle wasting in SARS-CoV-2 infected patients	Muscle Biopsy	Completed	NCT04698798	January 2021	Myalgia, elevated C-reactive protein, and cytokine
Respiratory muscle strength in volleyball players suffered from COVID-19	Respiratory function	Completed	NCT04789512	March 2021	COVID-19 players had considerably lower percent maximum inspiratory pressure and measured maximal expiratory pressure values than non-COVID-19 players. [124]
Relaxation exercise in patients with COVID-19	Relaxation technique	Completed	NCT04998708	August 2021	Positive effects on immune functions, progressive muscle relaxation

Abbreviations: LBM: lean body mass; WS: weight stability; QOL: quality of life; BW: body weight; WG: weight gain; Hb: Hemoglobin; PFS: progression-free survival; OS: overall survival.

## Abbreviations

SARS-CoV-2	severe acute respiratory syndrome- corona virus-2
SARS	severe acute respiratory syndrome
ACE2	angiotensin-converting enzyme 2
IL	interleukins
IF	interferons
TNF	tumor necrosis factors
TGF	transforming growth factors
CSF	colony-stimulating factors
LPL	lipoprotein lipase
ATGL	adipocyte triglyceride lipase
CRP	C- reactive protein
ICU	intensive care unit
CIF	cachexia- inducing factors
CRC	colorectal cancer
TAMs	tumor-associated macrophages
MCP1	monocyte chemoattractant protein 1
ACTRIIB	activin receptor type IIB
NFB	nuclear factor B
CHO	Chinese hamster ovary
CRH	corticotrophin-releasing hormone
TNFR1	TNF-α-receptor protein type I
ECM	extracellular matrix
RyR	ryanodine receptor5
LSMM	low skeletal muscle mass
SMM	skeletal muscle mass
CT	computed tomography
MERS	middle east respiratory syndrome
Th1	T helper 1
ARDS	acute respiratory distress syndrome
CRP	C-reactive protein
FDA	Food and Drug Administration
NSCLC	non-small cell lung carcinoma
MIC1	macrophage inhibitory cytokine 1
SNP	single-nucleotide polymorphisms
